# Malaria Vectors Insecticides Resistance in Different Agroecosystems in Western Kenya

**DOI:** 10.3389/fpubh.2018.00055

**Published:** 2018-03-01

**Authors:** Christine Ludwin Wanjala, Eliningaya J. Kweka

**Affiliations:** ^1^Department of Medical Laboratory Sciences, Masinde Muliro University of Science and Technology, Kakamega, Kenya; ^2^Department of Zoological Sciences, Kenyatta University, Nairobi, Kenya; ^3^School of Medicine, Catholic University of Health and Allied Sciences, Mwanza, Tanzania; ^4^Tropical Pesticides Research Institute, Arusha, Tanzania

**Keywords:** malaria, control, resistance, *Anopheles gambiae*, Ahero, Kisian, Emutete, Kabula

## Abstract

**Background:**

Malaria vector control efforts have taken malaria related cases down to appreciable number per annum after large scale of intervention tools. Insecticides-based tools remain the major control option for malaria vectors in Kenya and, therefore, the potential of such programs to be compromised by the reported insecticide resistance is of major concern. The objective of this study was to evaluate the status of insecticide resistance in malaria vectors in different agro ecosystems from western Kenya.

**Methods:**

The study was carried out in the lowlands and highlands of western Kenya namely; Ahero, Kisian, Chulaimbo, Emutete, Emakakha, Iguhu, and Kabula. World Health Organization tube bioassays was conducted using standard diagnostic dosages of Lambdacyhalothrin, Deltamethrin, Permethrin, DDT, Bendiocarb, and Malathion tested on *Anopheles* mosquitoes collected from seven sites; Ahero, Kisian, Chulaimbo, Emutete, Emakakha, Iguhu, and Kabula. Biochemical assays, where the enzymatic activity of three enzymes (monooxygenases, esterases, and glutathione *S*-transferases) were performed on susceptible and resistant mosquito populations. Wild mosquito populations were identified to species level using polymerase chain reaction (PCR). The species of the wild mosquito populations were identified to species level using PCR. Real-time PCR was performed on the susceptible and resistant mosquitoes after the WHO tube bioassays to determine the presence of knockdown resistance (*kdr*) allele.

**Results:**

WHO susceptibility tests indicated that *Anopheles gambiae* showed resistance to Pyrethroids and DDT in all the study sites, to Bendiocarb in Iguhu and Kabula and susceptible to Malathion (100% mortality) in all the study sites. There was an elevation of monooxygenases and esterases enzymatic activities in resistant *An. gambiae* mosquito populations exposed to Lambdacyhalothrin, Permethrin, Deltamethrin and DDT but no elevation in glutathione *S*-transferases. A high frequency of L1014S allele was detected in *An. gambiae* s.s. population, but there was no kdr allele found in *Anopheles arabiensis* mosquitoes.

**Conclusion:**

*An. gambiae* mosquitoes from western Kenya have developed phenotypic resistance to pyrethroids and DDT. Therefore, there is a need for further research covering different climatic zones with different agroeconomic activities for detailed report on current status of insecticide resistance in malaria vectors.

## Introduction

Malaria control programs in Sub Saharan Africa involve the use of Pyrethroid insecticides both in long-lasting insecticide nets (LLINs) and for indoor residual spraying (IRS) (1). Currently, malaria control mainly depends on pyrethroids, the only class of insecticide approved to be impregnated on mosquito nets, and it is also being widely used in IRS programs in Africa (2). Proper use of these malaria control tools can result in a remarkable reduction of morbidity and mortality associated with malaria (3, 4). There is hope of elimination of malaria in Africa as the usage of LLINs and IRS have proven to be effective in control of malaria (5). However, the recent emergence of mosquitoes to pyrethroids has become a major problem and, therefore, a threat to malaria vector control interventions.

Other classes of insecticides are available and have been used in some regions for malaria control. For instance, organophosphates have been used for IRS in form of Fenithrothion, Malathion, and Priphos-methyl in some countries (6). They have shown to be highly potent to mosquitoes, although they have a relatively short residual activity of 2–3 months when used for IRS (6). The mode of action of organophosphates involves inhibition of cholinesterase, stopping the breakdown of acetylcholine, which results in the overstimulation of the neuromuscular leading to the death of the vector (7). Carbamates, have been used in form of Bendiocarb and Propoxur, for IRS. They act the same way as organophosphates and, like organophosphates, they are very effective, but have a short residual activity (7).

There are two main mechanisms of insecticide resistance, which include: target site alteration, which reduces the capability of the insecticide to bind to the insect, thus lowering the amount of the insecticide reaching the insect (8, 9). This type of resistance is exhibited by knockdown resistance (kdr) to pyrethroids and DDT, which is caused by mutation in the voltage-gated sodium channel (VGSC) (10). L1014F-*kdr* mutation is mainly found in *Anopheles gambiae* from West Africa where as L1014S-kdr mutation mainly seen in *An. gambiae* from East Africa regions (11). Resistance to Organophosphates and Carbamates is caused by Alteration of target site in *An. gambiae* single amino acid substitution of glycine to serine at position 119 in the catalytic domain of the acetylcholinesterase gene (12). The second mechanism of resistance involves metabolic resistance, which occurs when there is elevation in metabolic enzyme activities, which detoxifies insecticides before binding to the target site (13). Monooxygenases are the major group of enzymes responsible for pyrethroid metabolism in insects (14).

Previous studies have reported an increase in pyrethroid resistance in East Africa; Ethiopia, Tanzania, Sudan, Uganda, and Kenya (9, 15–21). Pyrethroid resistance in malaria vectors has been reported in Kenya (22–29). With wide spread resistance to pyrethroids and DDT, carbamate and organophosphate classes of insecticides are the possible alternatives that can be considered for malaria control. Few studies have addressed the patterns of insecticide resistance in different agro ecosystems and insecticide susceptibility profile to the available alternative insecticides such as organophosphates and carbamates for malaria control. The objective of this study was to assess the status of *An. gambiae* resistance to pyrethroids, carbamates, and organophosphates in the highlands and low lands of western Kenya.

## Materials and Methods

### Study Sites

**Figure 1:**
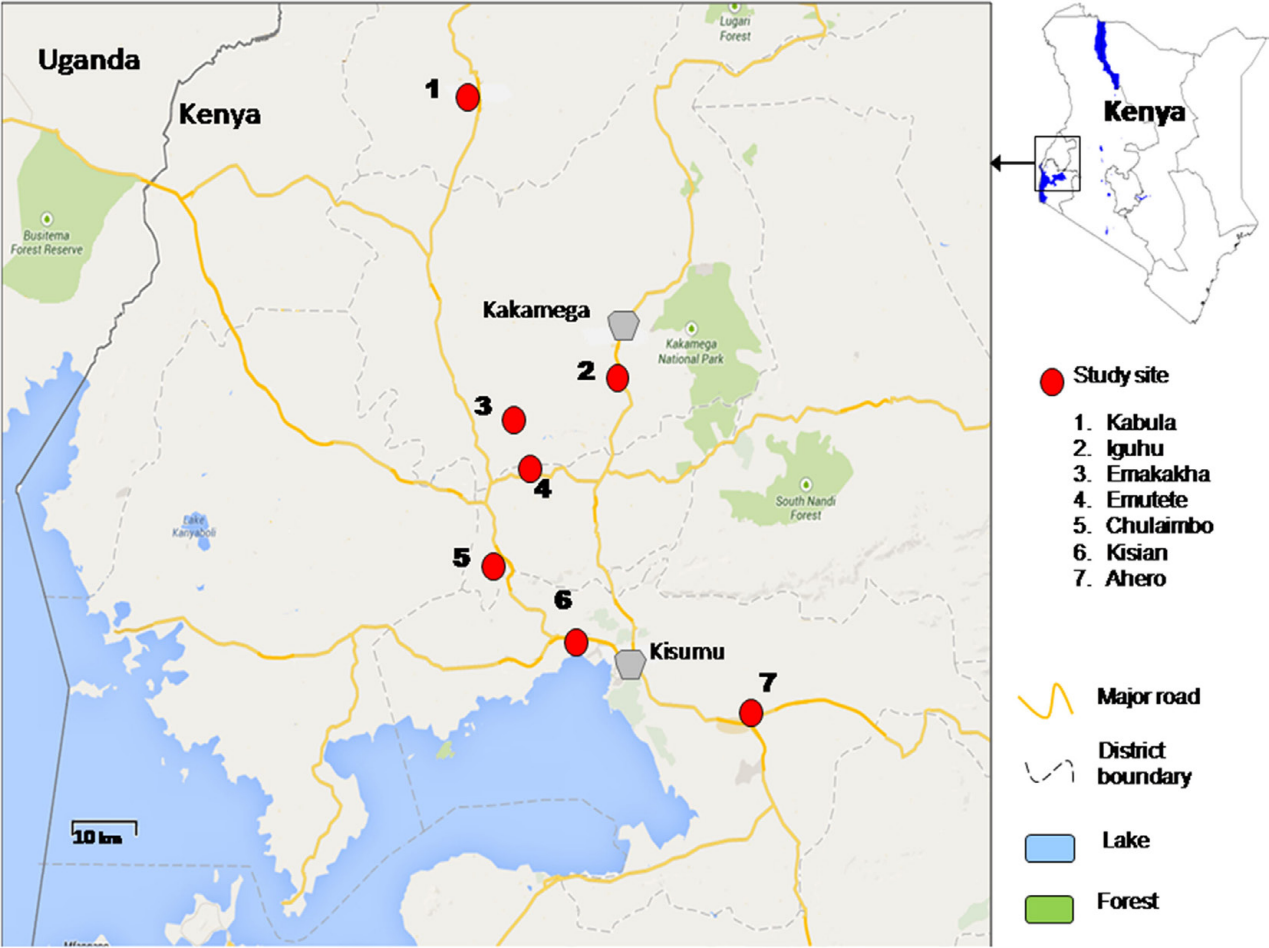
Map of study sites in western Kenya.

The study was conducted in lowlands and highlands of Western Kenya namely Ahero, Kisian, Chulaimbo, Emutete, Emakakha, Iguhu, and Kabula. Ahero (00.17259°S, 034.91983°E, altitude 1,162–1,360 m above the sea level), Chulaimbo (00.03572°S, 034.62196°E, altitude 1,328–1,458 m above the sea level) and Kisian (00.02464°S, 033.60187°E, altitude 1,280–1,330 m above the sea level) are located in Kisumu County (Low lands); Emutete (34°64 E, 00°22°N, elevation 1,463–1,603 m above the sea level), and Emakakha (34°64′E, 0°22′N, 1,463–1,604 m above sea level), are found in Vihiga County (highlands); Iguhu (0°17′N, 34°74′E, and elevation 1,450–1,580 m above sea level) is located in Kakamega County (highland) and Kabula (00.54057°N, 034.56410°E, altitude 1,545 m above the sea level) is located in Bungoma County, in highland area (Figure 1).

Malaria transmission is perennial in the lowland sites of Ahero, Kisian, and Chulaimbo. *An. gambiae sensu strict (*s.s.*)* and *Anopheles arabiensis* are both found in lowland sites (27) except Ahero, where only *An. arabiensis* has been collected since the late 1990s (21). The highland sites of Emutete, Emakakha, Iguhu, and Kabula have low seasonal malaria transmission, with peaks at the end of the long (early April to early June) and short (October–November) rainy seasons and high year-to-year variation. The prevalent malaria vector in the highlands is *An. gambiae s.s*. (27) and others in minority are *Anopheles funestus* (30, 31) and *An. arabiensis* (32).

### Ethical Clearance

Ethical clearance was obtained from the Ethical Review Board at the Kenya Medical Research Institute and University of California, Irvine (SSC no 1382). Our study activities were authorized by the area chiefs, subchiefs and village elders. The permission to collect mosquito from the fields was obtained from the field owners. Our study did not pose any danger to the communities involved.

### Mosquito Larvae Sampling

*Anopheles* mosquito larvae were collected from various aquatic habitats using standard dipper (350 ml) and pipettes. The samples were then transported to an insectary within KEMRI, Kisumu. They were then transferred to rearing pans and fed on a mixture of fish food and brewer’s yeast that was changed daily. After pupation, the pupa was transferred to cages where they were allowed to emerge to adults. The adult mosquitoes were fed on 10% sucrose in cotton swabs.

### WHO Tube Bioassays

Two to five days old unfed female mosquitoes collected from seven study sites (Kabula, Iguhu, Emutete, Emakakha, Ahero, Chulaimbo, and Kisian) were used to test for the susceptibility to the following insecticides; Lambdacyhalothrin (0.05%), deltamethrin (4%), permethrin (0.75%), DDT (0.05%), malathion (5%); and bendiocarb (0.1%). Kisumu strain, a reference mosquito insectary susceptible colony reared at KEMRI was used as control. Mosquitoes were released and kept in the exposure tubes for 60 min and then transferred back to the paper cups. They were then maintained in the paper cups for 24 h (to allow them to recover) where they were supplied with a pad of a cotton-wool soaked in 10% sugar solution. The mortality of the mosquitoes was scored immediately after 24 h, and the susceptibility status of the mosquito populations graded according to the WHO standard (33). A total of 200 mosquitoes were tested per site per insecticide. Tests were carried out at 24–26°C and 80–90% relative humidity during the 1-h exposure period and the subsequent 24-h holding period, with a 12D:12N photophase. Susceptible and resistant mosquitoes from this bioassay were separately kept in RNA later solution at −20°C for species identification and molecular characterization.

### Biochemical Assays

Expression of metabolic enzymes and total protein was measured using the microplate enzyme system protocol developed by Brogdon and others (34). Freshly collected mosquitoes were used for these assays from all seven study sites after exposure to WHO insecticide impregnated filter papers (35).

### Species Identification Using Polymerase Chain Reaction (PCR)

The methodology developed by Scott and others was used for *Anopheles* species identification of mosquitoes exposed to WHO tube bioassays (36). DNA was extracted using ethanol precipitation from legs and wings (37). The two sibling species (*An. gambiae* s.s. and *An. arabiensis*) of the *An. gambiae s.l*. species complex were distinguished using conventional PCR (36).

### Genotyping Using Real-time PCR

The presence of kdr gene in the mosquitoes preserved after the WHO bioassays was detected using real-time PCR. The DNA extraction was conducted using the protocol established by Collins and others (37). The genotyping of amino acid position 1014 of the VGSC was done using, following the methods of Bass et al (38) and its modification done by Mathias and others (39).

### Data Analysis

The mortality of mosquitoes after WHO tube resistance bioassay was calculated using the formula below (40):

Total number of susceptible mosquitoes/Total number of exposed mosquitoes∗100

Susceptibility status of a mosquitoes was classified using the WHO criteria (98–100% mortality indicates susceptibility, 90–97% mortality suggests possibility of resistance that needs to be confirmed, and <90% mortality suggests resistance) (35). The data were labeled by the site of origin. The mortality of mosquitoes was compared between different sites and between different insecticides using ANOVA. The fold increase in the enzymatic activity was calculated by dividing the optical density of the resistant mosquitoes by that of susceptible mosquitoes from the same site and was done separately for all sites. of the frequency of *kdr* allele in different mosquito populations was calculated. To determine if these genotypes were under selection, Hardy–Weinberg equilibrium test for *kdr* genotypes was performed, and χ^2^ test was used to determine the significance of the departure from Hardy–Weinberg equilibrium.

## Results

### The Status of Phenotypic Resistance of the Mosquito Population Studied

Kisumu Strain, Ahero, Emakakha, and Emutete were highly susceptible to Bendiocarb with 100% mortality but did not differ significantly from Kisian and Chulaimbo mosquitoes, which had 99 and 98% mortality, respectively. Iguhu (87%) and Kabula (84%) mosquito populations had a significantly lower mortality compared to the other sites and also differed significantly (*F* = 92.89, *P* = 0.0001).

**Figure 2:**
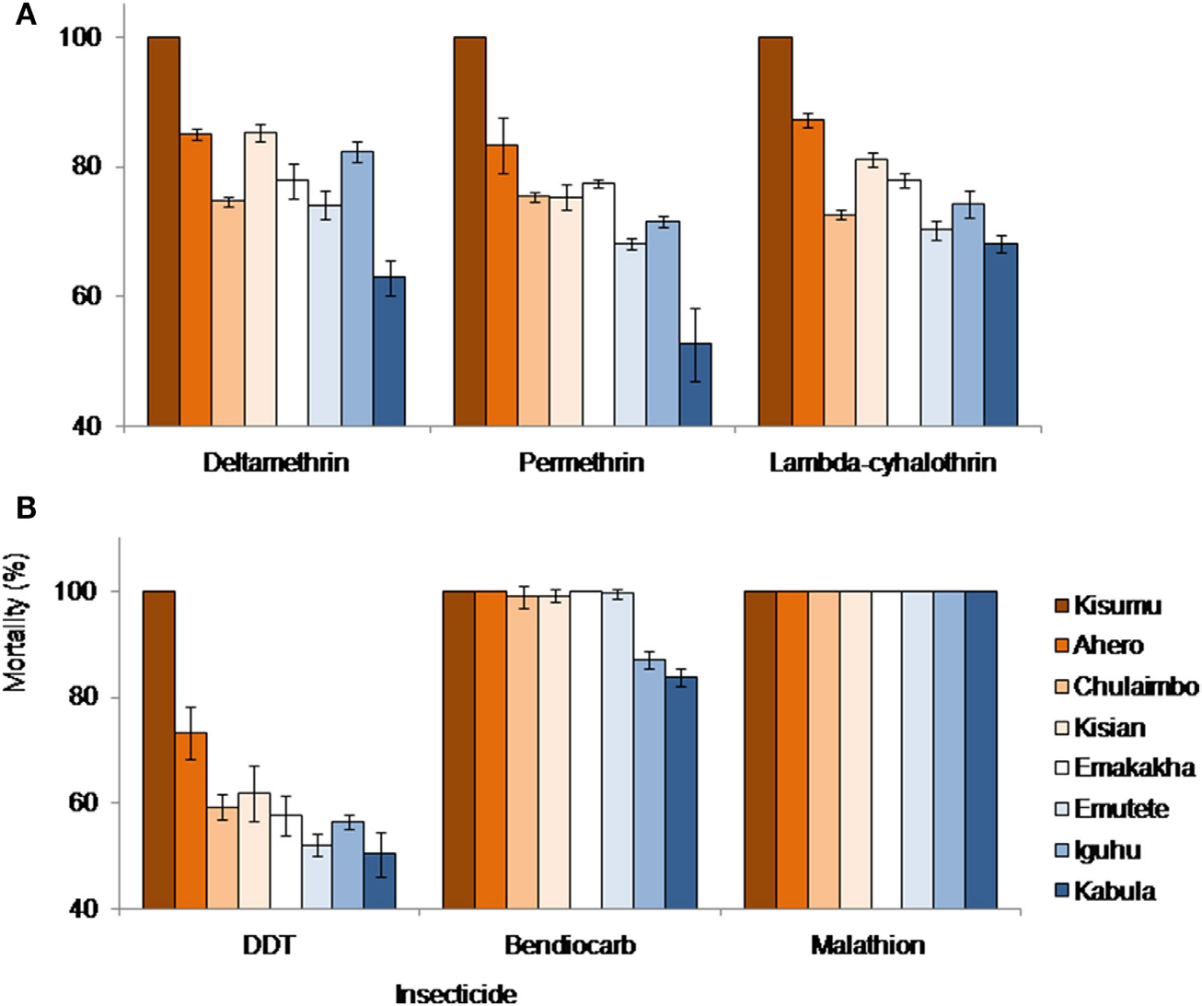
*Anopheles gambiae s.l*. mortalities against different insecticides and study sites. **(A)** Mortalities against different formulation of pyrethroid insecticides: deltamethrin, permethrin, and lambdacyhalothrin; **(B)** mortalities against DDT (Organochlorine), bendiocarb (Carbamate), and Malathion (Organophosphate). Kisumu strain was used as control.

All mosquito populations showed remarkable resistance to DDT except Kisumu strain (Insectary colony) that showed 100% mortality. Ahero mosquito populations had significantly higher mortality (73%) compared to the other sites (*F* = 16.70, *P* < 0.0001). There were no significant differences between the mortality of the mosquitoes from Emakakha, Emutete, Iguhu, Kisian, and Chulaimbo. The lowest mortality was recorded in Kabula mosquito population, which was significantly different from Kisian mosquitoes, but did not differ from Emakakha, Emutete, Iguhu, and Chulaimbo mosquitoes (Figure 2).

Mosquitoes from all sites were resistant to lambdacyhalothrin (mortality <90%) except Kisumu strain that showed 100% mortality. Ahero mosquitoes had a significantly higher mortality compared to other sites, followed by Kisian mosquitoes, which were significantly different from Emakakha mosquitoes (*F* = 62.05, *P* = 0.0001). The mortality of Chulaimbo and Iguhu mosquitoes did not differ significantly. Kabula mosquitoes had the lowest mortality but did not differ significantly from Emutete and Chulaimbo mosquitoes.

When exposed to diagnostic dosage of Deltamethrin, Kisumu strain had a significantly high mortality compared to other sites, followed by the mortality of mosquitoes from Kisian mosquitoes, which did not differ significantly from the mortality of Ahero and Iguhu mosquitoes. There was no significant difference in the mortality of Emutete, Emakakha, and Chulaimbo mosquitoes. The mortality of Kabula mosquitoes was significantly lower than all the other sites (*F* = 55.65, *P* = 0.0001). Mosquitoes from all sites were resistant to Deltamethrin except Kisumu strain that was used as a control (Figure 2).

Finally, when exposed to Permethrin, the mortality of mosquitoes from Kisumu strain had significantly higher mortality compared to the other sites. The mortality of mosquitoes from Ahero significantly different from the mortality of Kisian, Chulaimbo and Emakakha mosquitoes. The mortality of Iguhu mosquitoes was not significantly different from that of Emutete mosquitoes. The mortality of Kabula mosquitoes was significantly lower compared to the other sites (*F* = 30.10, *P* = 0.0001). Mosquitoes from all the study sites except Kisumu strain were resistant to Permethrin (Figure 2).

### Biochemical Assays

#### Metabolic Activity of Monooxygenases (P450)

**Figure 3:**
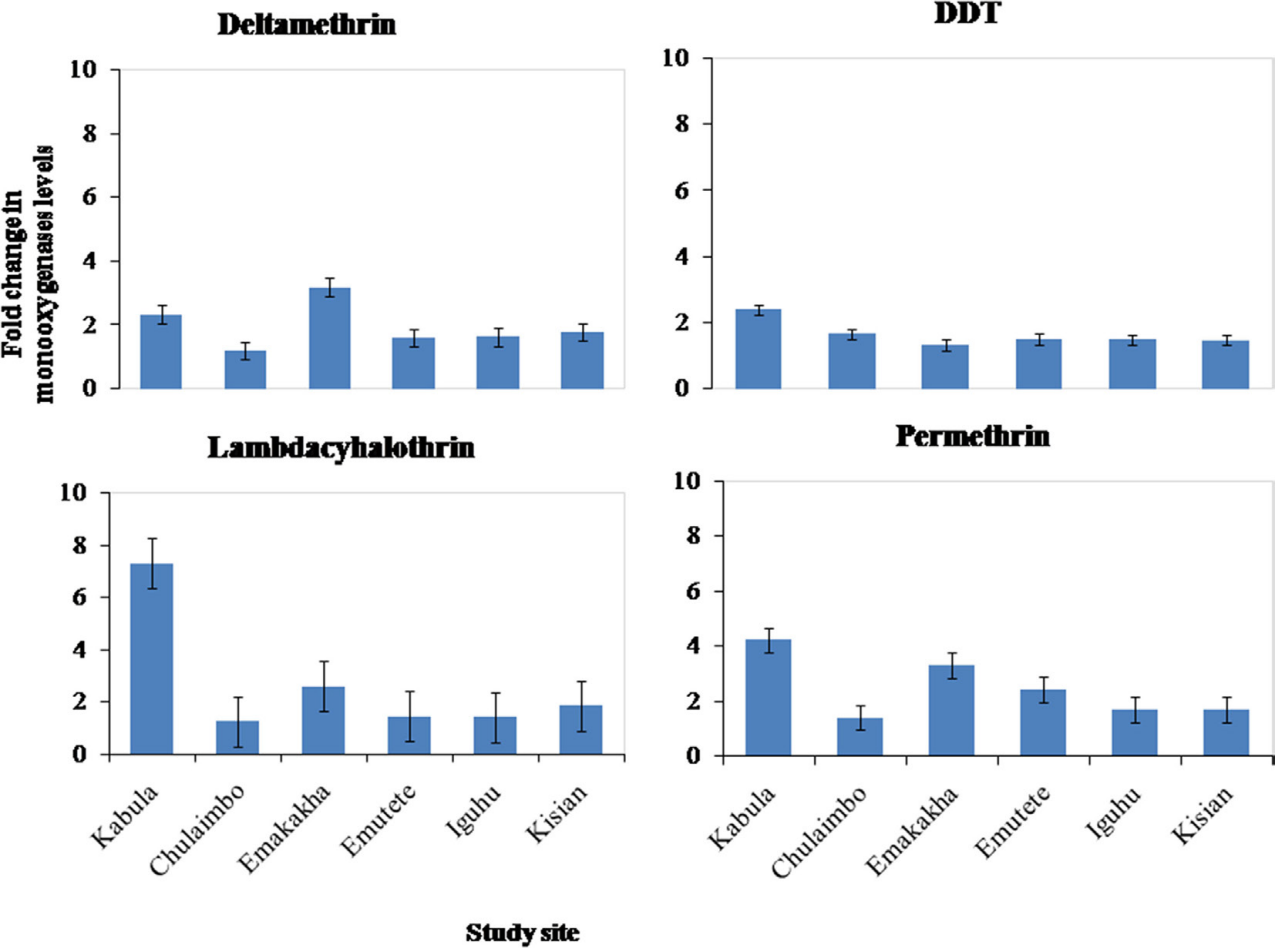
Fold change in the monooxygenases (P450) levels (±SE) in resistant mosquitoes against susceptible mosquitoes from the same study site.

There was an increase in the monooxygenases enzymatic activity in resistant mosquitoes exposed to Pyrethroids (Permethrin, lambdacyhalothrin, Deltamethrin) and DDT. There was a sevenfold increase in the monooxygenases enzymatic activity in Kabula mosquito populations exposed to lambdacyhalothrin, 2.5 in Emakakha, 1.8 Emutete populations, and 2 in Iguhu populations, but there was no increase in the monooxygenases activity in Kisian and Chulaimbo-resistant mosquitoes exposed to Lambdacyhalothrin (Figure 3).

There was a 4- and 2.5-fold in Kabula mosquito populations exposed to permethrin and deltamethrin, respectively, 3.8- and 3.6-fold in Emakakha populations, 3- and 2-fold in Emutete populations, 2- and 2-fold in Iguhu populations, and finally 2- and 2-fold increase in Kisian populations. The monooxygenase enzyme activity increased in resistant mosquito populations exposed to DDT in Kabula mosquito populations and there was a 2.5-fold increase, in Chulaimbo 1.8-fold increase, 1.7-fold increase in Emakakha, Emutete, Iguhu, and Kisian mosquito populations (Figure 3).

#### Metabolic Activity of Esterase Activity

**Figure 4:**
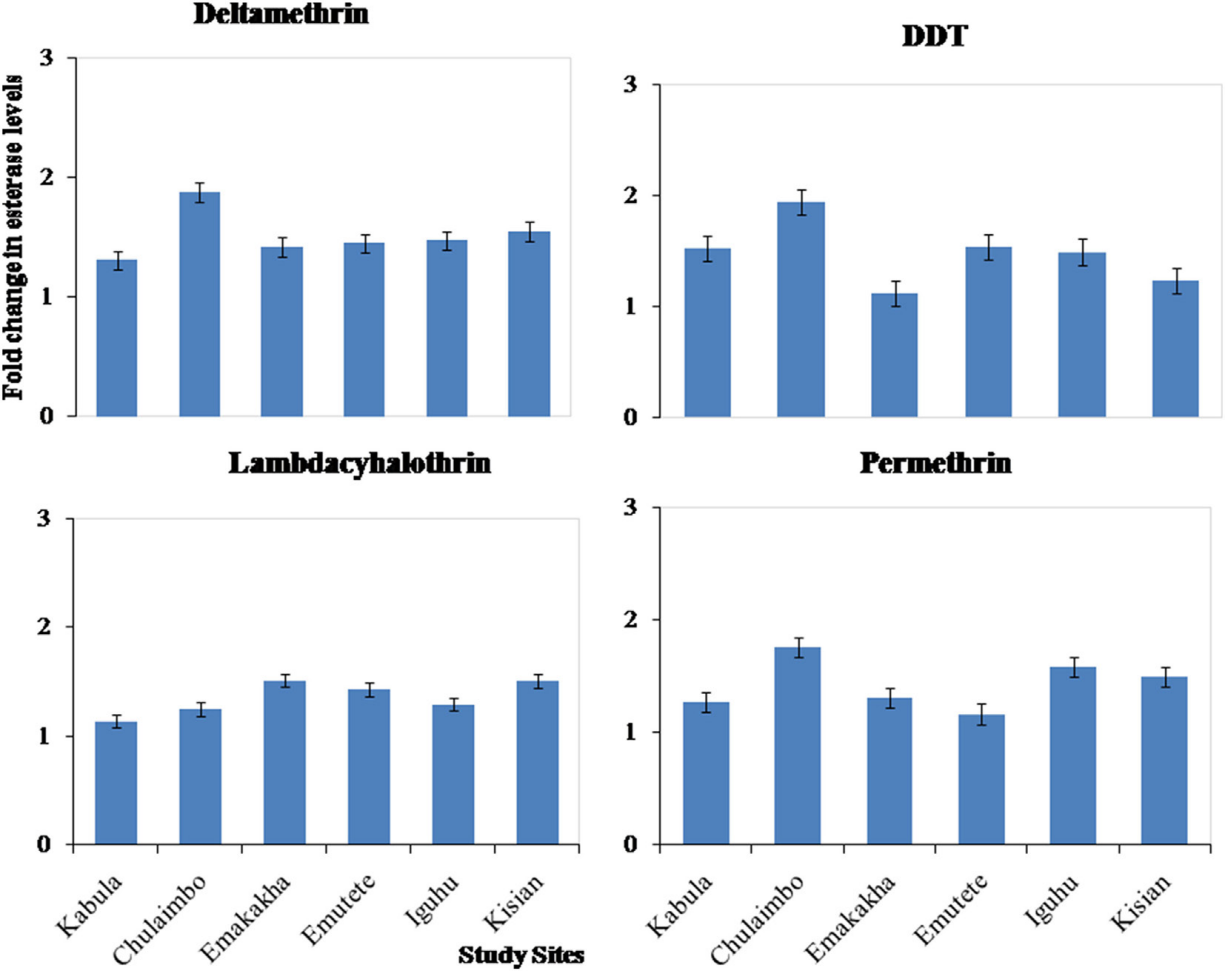
Fold changes in esterase levels (±SE) in resistant mosquitoes against susceptible mosquitoes from the same study site.

There was also an increase in esterases activity in resistant mosquitoes exposed to pyrethroids (permethrin, lambdacyhalothrin, and deltamethrin) and DDT. There was a 1.1-, 1.2-, and 1.2-fold increase in Kabula mosquito populations exposed to Lambdacyhalothrin, permethrin, and deltamethrin, respectively, 1.2-, 1.8-, and 2-fold increase in Chulaimbo mosquito populations, 1.5-, 1.3-, and 1.5-fold increase in Emakakha populations, 1.5-, 1-, and 1.6-fold increase in Emutete populations, 1.4-, 1.7-, and 1.6-fold in Iguhu populations and finally 1.5-, 1.6-, and 1.5 in Kisian mosquito populations. There was also an increase in esterases activity in resistant mosquitoes exposed to DDT, 1.2-fold increase Kabula, Emutete, Iguhu, and Emutete mosquito populations, 1.5-fold increase in Chulaimbo populations, 1-fold increase in Kisian, and 0.7-fold increase in Emakakha mosquito populations (Figure 4).

#### Glutathione-*S*-Transferase Enzymatic Activity

**Figure 5:**
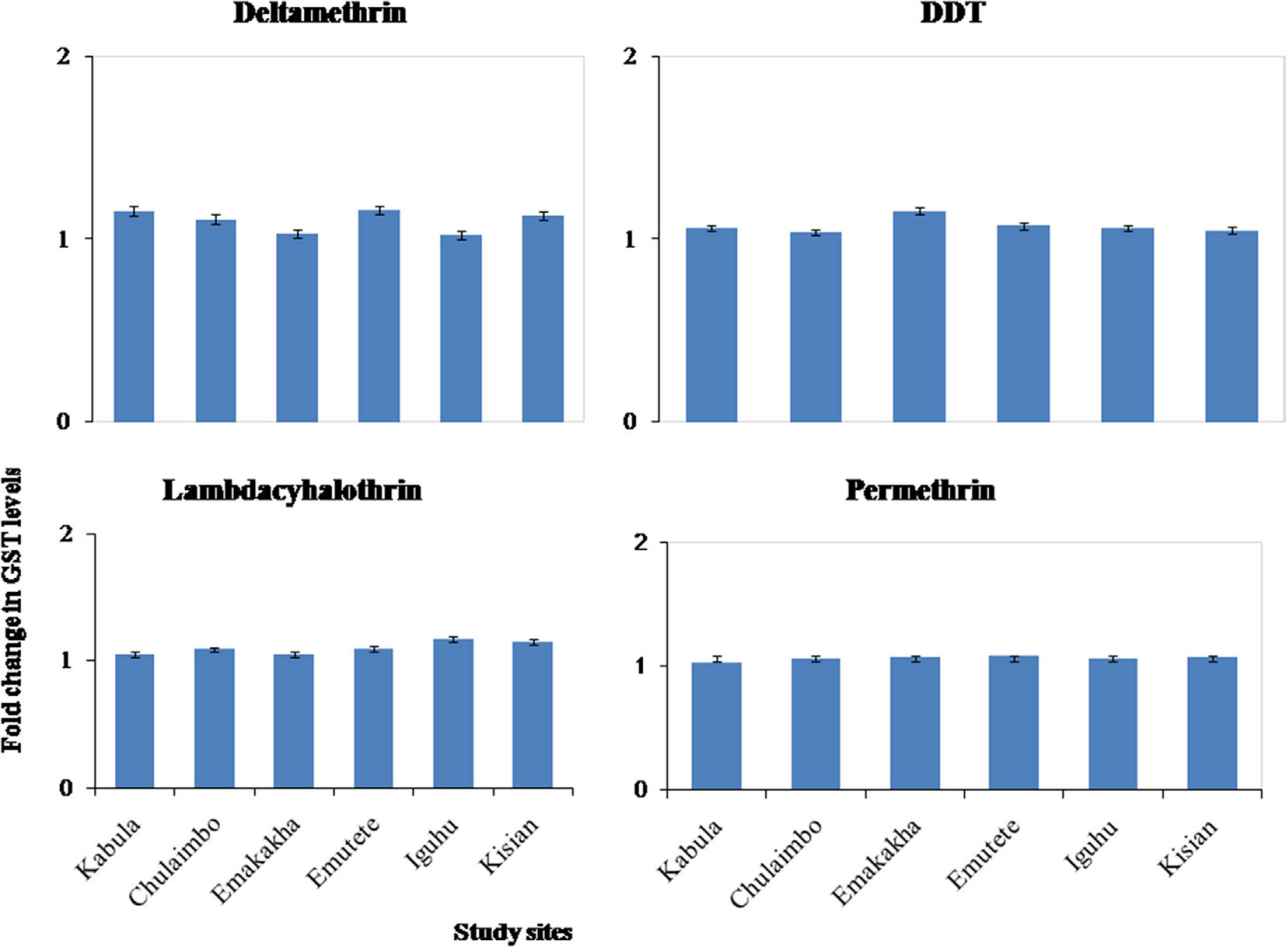
Fold changes in GST levels (±SE) in resistant mosquitoes against susceptible mosquitoes from the same study site.

There was onefold change in the GST activity in the resistant mosquitoes that were exposed to pyrethroids and DDT in all the study sites (Figure 5).

#### Species Identification

Polymerase chain reaction results indicated that *An. gambiae* was the predominant species in Chulaimbo (72%), Emutete (94.0%), Emakakha (93.4%), Iguhu (88.0%), and Kabula (90.0%), where as *An. arabiensis* was predominant in Kisian (64.4%) and Ahero (89.3%) (Table 1).

**Table 1:** Species composition at different study sites.

Study site	*N*	*Anopheles arabiensis* (%)	*Anopheles gambiae* s.s. (%)	Not determined (%)
Ahero	56	89.3	5.4	5.4
Kisian	225	64.4	32.9	2.4
Chulaimbo	100	24.0	72.0	4.0
Emutete	200	3.5	94.0	2.5
Emakakha	61	3.3	93.4	3.3
Iguhu	300	8.0	88.0	4.0
Kabula	60	3.3	90.0	6.7

#### *Kdr* Allele Genotyping

Homozyzygous *kdr* (L1014S) was high in *An. gambiae* s.s. populations, but relatively low in *An. arabiensis* dominant populations. The frequency of *kdr* allele was significantly high in Chulaimbo and Emakakha, followed by Emutete, Iguhu, and Kabula. The mosquitoes from Kisian had the lowest frequency of kdr allele in the *An. gambiae* mosquitoes. In *An. arabiensis* mosquitoes, the frequency of the kdr allele was high in Chulaimbo mosquitoes and Bungoma mosquitoes followed by Ahero, Iguhu, and finally Kisian. There was no mutation in the *An. arabiensis* mosquitoes from Emakakha. *Kdr* genotyping was not successful in 2.8% of the samples and mutations were observed in *An. gambiae* were homozygous. Only one population (Chulaimbo) out of the four *An. arabiensis* populations tested showed significant deviation that resulted from heterozygosity deficiency. On the other hand, five out of the six *An. gambiae* populations tested showed significant departure from Hardy–Weinberg equilibrium, all caused by heterozygosity deficiency (Table 2).

**Table 2:** Genotype and allele frequencies of knockdown resistance (*kdr*) at the seven study sites in western Kenya.

Study site	*Anopheles gambiae*						
	*N*	LL	LS	SS	Frequency	χ^2^	*P*-value
Ahero	3	3	0	0	0.0	–	–
Kisian	50	32	3	15	30.0	37.35	<0.0001
Chulaimbo	56	2	4	50	89.3	11.93	<0.0001
Emutete	87	7	4	76	87.3	49.22	<0.0001
Emakakha	57	1	7	49	85.9	1.38	0.24
Iguhu	108	10	7	91	84.3	53.48	<0.0001
Kabula	53	5	5	43	81.1	19.83	<0.0001

	***Anopheles Arabiensis***						
	***N***	**LL**	**LS**	**SS**	**Frequency**	**χ^2^**	***P*-value**

Ahero	50	46	4	0	0	0.09	0.77
Kisian	42	41	1	0	0	0.01	0.94
Chulaimbo	23	14	0	9	39.1	23.00	<0.0001
Emutete	0	0	0	0	0	–	–
Emakakha	1	1	0	0	0.0	–	–
Iguhu	16	15	1	0	3.1	0.02	0.90
Kabula	3	2	0	1	33.3	–	–

*N is sample size, LL represents wild type, LS represents heterozygote mutation, SS represents homozygote mutation,” and frequency is the mutation allele frequency (%). Symbol “–” stands for not done. χ^2^ and P-value are the results of Hardy–Weinberg equilibrium test*.

## Discussion

Effective insecticide-based malaria vector control depends on the geographical location of the vectors and type of the insecticide used. Results from this study showed a marked variation of mortality of mosquitoes from different sites exposed to different classes of insecticide. The resistance of mosquitoes to insecticides that was observed in the study was brought about partly by elevation of metabolic enzymes and the presence of the resistance allele *kdr* in resistant mosquitoes.

Results from this study indicate that Malathion was highly potent to mosquitoes recording a mortality of 100% in all the study sites (Table 1). The mortality of mosquitoes from five study sites (Ahero, Kisian, Chulaimbo, Emutete, and Emakaha) was high when exposed to Bendiocarb, but resistance was observed in two sites, Iguhu and Kabula, when they were exposed to the same insecticide (Table 2). This suggests that mosquitoes in western Kenya have not yet developed resistance to Malathion and Bendiocarb. Mosquitoes from all the study sites showed marked resistance to DDT recording the highest mortality of 78% in Ahero mosquitoes. When exposed to pyrethroids (lambdacyhalothrin, permethrin, and deltamethrin), all mosquito populations showed resistance with the highest mortality of 87% observed in Ahero populations exposed to Lambdacyhalothrin. The difference in the mortality rates within different insecticide can be explained by the difference in the mode of actions, Malathion and Bendiocarb inhibit cholinesterase whereas pyrethroids and DDT are modulators of voltage gated sodium channels (7). The observed resistance of mosquitoes to pyrethroids can be caused by over use of this insecticide of malaria control in Kenya for control of Malaria, which has led to cross resistance in DDT, because the these classes of insecticides have the same similar mode of action (8).

The results from this study coincides with previous studies by Chouaibou et al. (41) who reported that *An. gambiae* populations in northern Cameroon were susceptible to carbamates and organophosphates but highly resistant to organochlorines and pyrethroids. However, they do not contradict the findings of Matowo et al. (42) who reported that *An. arabiensis* mosquito populations in Tanzania were highly susceptible to organochlorine (DDT), organophosphate, and carbamate, but highly resistant to pyrethroids. The observed pyrethroid resistance in western Kenya could be associated with massive scale up of insecticide-based vector control in Kenya and extensive use of this group of pesticide to control agricultural pests (28, 39).

Previous studies in Kenya reported that the localized use of insecticide-treated nets in Kisumu increased the permethrin tolerance of *An. gambiae* populations (43, 44). Stump et al. (23) also observed an increase in the level of mosquitoes to pyrethroids in western Kenya as a result of massive scale up of ITN distribution and usage. In Moshi, Tanzania, a field trial by Mosha et al. (45) reported that while commonly used ITNs killed relatively few host-seeking *An. arabiensis*, the nets continued to provide personal protection through the strong excito-repellent activity of permethrin (45). Kawada et al. (46) reported that resistant *An. gambiae* populations from Kenya were tolerant to excito-repellent activity of LLINs. However, LLINs have been reported to be still effective in malaria control, and the effect of insecticide resistance to malaria control has not been documented (47).

Results from this study indicated a significant difference in mortality of mosquitoes from different sites when exposed to Bendiocarb, Pyrethroids, and DDT. Ahero Mosquitoes recorded the highest mortality followed by Kisian, where as the mean mortality of mosquitoes from Emutete, Emakakha, and Iguhu did not differ significantly when exposed to the insecticides. Kabula Mosquito populations showed the lowest mortality when exposed to all the insecticide except Malathion. This suggests that topographical features can have an effect of the mosquito survival and longevity, thus affecting their response to insecticides (48–50). The marked resistance observed in Kabula mosquito populations can be linked to the spread of the resistance alleles from Uganda, since this region borders Uganda where insecticide resistance to pyrethroids is widespread (16, 19). The same observation was made by Ochomo et al. (27) who reported resistance in Bungoma mosquitoes and high susceptibility in Ahero Mosquitoes.

This study also reported the elevation of monooxygenases and esterases enzyme activity in resistant *An. gambiae* mosquito populations from Kabula, Iguhu, Emutete, Emakakha and Chulaimbo and Kisian exposed to Permethrin DDT, but no elevation of monooxygenases and esterases in *An. arabiensis* mosquitoes from Ahero. There was no elevation of GSTs in all mosquito populations. This suggests that the observed resistance to permethrin and DDT these areas could be caused by elevation of the enzyme activity of monooxygenases and esterases, but not the GSTs. For instance, mosquitoes from Kabula had the lowest mortality when exposed to pyrethroids and DDT, there was the highest fold change (eightfold) in the monooxygenase enzyme activity in the same mosquito population, when they were exposed to DDT. The lack of elevation in monooxygenases and esterase enzyme activity in Ahero mosquitoes could explain why the mosquitoes had the highest mortality when exposed to the same insecticides. In a similar study, Matowo et al. (42) eluded the observed pyrethroids and DDT resistance to the elevation of metabolic enzyme activities in the mosquito populations. The results from this study coincides with the previous studies by Ochomo et al. (27) that reported an elevation in esterases and monooxygenases enzyme activities in *An. gambiae* permethrin-resistant populations from Bungoma and Budalangi. Vulule et al. (43, 44) reported an elevation in both β-esterase and oxidase enzyme expression in *An. gambiae* permethrin-resistant populations, but there was no elevation in GST enzymes levels in the *An. gambiae* s.s. Furthermore, no elevation of monooxygenases enzyme activities was observed in *An. arabiensis* populations from Ahero even though this area had previously been covered by a pyrethroid-based IRS and LLINs. This contradicts the previous reports that extensive use of insecticide-based vector control methods could be selected for resistance in malaria vectors (23).

There was a marked phenotypic resistance to deltamethrin and lambdacyhalothrin in this study, but no measurable elevated expression of monooxygenases and esterases in resistant mosquitoes that had been exposed to deltamethrin and lambdacyhalothrin. This suggests that mechanisms of resistance to Deltamethrin and Lambdacyhalothrin could be due to target site alteration as a result of the presence of the *kdr* alleles, whereas resistance against permethrin and DDT may involve target site resistance and metabolic resistance as a result of the elevation of β-esterase and monooxygenase enzymes activities (8).

Results of species identification indicated that *An. gambiae* was the predominant species in Kabula, Iguhu, Emutete, Emakakha, and Chulaimbo whereas *An. arabiensis* is predominant in Ahero and Kisian. Lack of *An. gambiae s.s*. in Ahero mosquito populations could be explained by *An. gambiae s.s*. species shift to zoophilic *An. arabiensis* following scaling-up of ITNs as reported earlier by Mathias et al. (39). Previous studies in these sites have reported the abundance of *An. gambiae* in Bungoma, Emutete, and Iguhu (51) and *An*. *funestus* in Kisian and Chulaimbo (52).

The findings from this study also showed that the frequency of kdr allele was high in *An. gambiae* from Kabula, Emutete, and Emutete mosquito populations. This could be due to extensive insecticide-based vector control activities in this regions especially IRS with deltamethrin and lambdacyhalothrin, and use of LLINs. kdr mutations in *An. gambiae* s.s. has been well documented (27, 46, 53, 54). Compared to *An. gambiae* s.s., kdr mutations in *An. arabiensis* was low in the study areas (mainly Ahero and Kisian), although WHO bioassay test did show that *An. arabiensis* were resistant to DDT and pyrethroid insecticides. This suggests that other insecticide-resistant mechanisms could be present in these mosquitoes. However, previous study by Kawada et al. (46) reported the presence of kdr alleles in *An. Arabiensis*, which is in contrast to the findings from this study (46). In Ahero, rice is the predominant crop and pesticides have been frequently used for pest control in Ahero even before the scaling-up of ITNs, but the kdr mutation at L1014S rate was found to be low. The agricultural use of pesticide types are different from the insecticides commonly used for IRS and ITNs in the area, this mixed use of multiple type of insecticides may have delayed the development of resistance in *An. arabiensis*. The increase in frequency of the 1014S *kdr* allele in western Kenya populations reported in a previous studies (39) and confirmed by this study indicate that the insecticide resistances is spreading and gradually increasing in the mosquito populations. Therefore, there is need for development and usage of non insecticide-based ecological tools such as house modification and improved larval control technologies, which may contribute to new intergrated vector management strategies, to mitigate against further spread of insecticide resistance.

### Study Limitation

The end point was not prespecified during study design, and the control of overall type I error rate was not possible during data analysis using the *post hoc* tests used. Therefore, the study was not adjusted for multiple hypothesis testing.

## Author Contributions

CW participated in the study design, performed laboratory work, data collection, data analysis, and drafted the manuscript. EK participated in study design and contributed to manuscript writing. Both authors agreed upon submission of this manuscript.

## Conflict of Interest Statement

The authors declare that the research was conducted in the absence of any commercial or financial relationships that could be construed as a potential conflict of interest.
